# Cell-Penetrating CaCO_3_ Nanocrystals for Improved Transport of NVP-BEZ235 across Membrane Barrier in T-Cell Lymphoma

**DOI:** 10.3390/cancers10020031

**Published:** 2018-01-25

**Authors:** Viviana Vergaro, Monica Civallero, Cinzia Citti, Maria Cosenza, Francesca Baldassarre, Giuseppe Cannazza, Samantha Pozzi, Stefano Sacchi, Francesco Paolo Fanizzi, Giuseppe Ciccarella

**Affiliations:** 1 Dipartimento di Scienze e Tecnologie Biologiche e Ambientali, Università del Salento & UdR INSTM di Lecce, Campus Universitario, Via Monteroni, 73100 Lecce, Italy; francesca.baldassarre@unisalento.it; 2 Dipartimento di Medicina Diagnostica, Clinica e di Sanità Pubblica, Università di Modena & Reggio Emilia, via Campi 287, 41125 Modena, Italy; monica.civallero@unimore.it (Mo.C.); maria.cosenza@unimore.it (Ma.C.); samantha.pozzi@unimore.it (S.P.); stefano.sacchi@unimore.it (S.S.); 3 Dipartimento di Scienze della Vita, Università di Modena e Reggio Emilia, Via Campi 103, 41125 Modena, Italy; cinzia.citti@unimore.it (C.C.); giuseppe.cannazza@unimore.it (G.C.); 4 CNR NANOTEC—Istituto di Nanotecnologia c/o Campus Ecotekne, Università del Salento, Via Monteroni, 73100 Lecce, Italy; 5 Dipartimento di Scienze e Tecnologie Biologiche e Ambientali, Università del Salento, Via Monteroni, 73100 Lecce, Italy; fp.fanizzi@unisalento.it

**Keywords:** CaCO_3_ nanocrystals, NVP-BEZ235, mass spectrometry, T-cell lymphoma, drug delivery, nanomedicine

## Abstract

Owing to their nano-sized porous structure, CaCO_3_ nanocrystals (CaCO_3_NCs) hold the promise to be utilized as desired materials for encapsulating molecules which demonstrate wide promise in drug delivery. We evaluate the possibility to encapsulate and release NVP-BEZ235, a novel and potent dual PI3K/mTOR inhibitor that is currently in phase I/II clinical trials for advanced solid tumors, from the CaCO_3_NCs. Its chemical nature shows some intrinsic limitations which induce to administer high doses leading to toxicity; to overcome these problems, here we proposed a strategy to enhance its intracellular penetration and its biological activity. Pristine CaCO_3_ NCs biocompatibility, cell interactions and internalization in in vitro experiments on T-cell lymphoma line, were studied. Confocal microscopy was used to monitor NCs-cell interactions and cellular uptake. We have further investigated the interaction nature and release mechanism of drug loaded/released within/from the NCs using an alternative approach based on liquid chromatography coupled to mass spectrometry. Our approach provides a good loading efficiency, therefore this drug delivery system was validated for biological activity in T-cell lymphoma: the anti-proliferative test and western blot results are very interesting because the proposed nano-formulation has an efficiency higher than free drug at the same nominal concentration.

## 1. Introduction

The main limit of anticancer drugs is the crossing of cell membranes; in particular, hydrophobic drug prefer to stay in the double lipid layer, whilst the polar drug molecules prefer to reach the aqueous cytoplasm environment. Furthermore, it’s known that low concentrations of drugs in the target tissues will lead to suboptimal therapeutic effects and require more frequent administrations; on the other hand, high drug concentrations might not be tolerated and could cause toxicity [1].

In this regard, the advent of nanomaterials provides an efficient mechanism for more convenient, controlled and targeted drug delivery, which is interesting in terms of application in the biomedical field, since they have a large effectiveness [2].

The importance of drug delivery has increased over the past decades, and significant advances have been made in the development of novel technologies. In order to create an ideal nano-carrier, considerable effort has been spent to make “soft” drug carriers, such as liposomes, micelles, dendrimers, and biodegradable polymers [3,4]. Beside them, inorganic nanomaterials have many fundamental advantages due to the ease of synthesis and modification [5]. 

Here, the use of calcium carbonate nanocrystals (CaCO_3_ NCs), obtained according to our patented protocol [6], was suggested as template in order to encapsulate a poorly water-soluble drug, NVP-BEZ235. The special properties of this novel material have been used in the biomedical communities with the hope of developing a new drug delivery system for therapeutic treatment agents such as proteins, nucleic acids, and other biologically active molecules [7,8,9,10,11], owing to their reduced toxicity effects due to their small size, stable structures phase, specific surface area, chemical purity, safety, biocompatibility, pH-sensitivity and slow biodegradability [12,13,14]. Given the potential abilities of CaCO_3_NCs to reach the intracellular compartment, the potential application of CaCO_3_NCs is used for transport and delivery of drugs and antibodies without toxic effects. This material is considered as an ideal drug carrier thanks to the excellent biocompatibility, targeting action, and subcellular localization.

For example, in a recent work, CaCO_3_ NCs were proposed as a pH-responsive carrier for delivery of chemotherapeutics or nucleic acids because it is stable under neutral pH and would be decomposed into Ca^2+^ and CO_2_ under acidic pH [15,16]. Zhao et al. reported that CaCO_3_ nanospheres loaded with doxorubicin exhibited greatly enhanced therapeutic effects over the free drug towards tumor cells [17]. In another research work, core shell CaCO_3_ nanoparticles loaded with doxorubicin, were used for in vivo liver cancer chemotherapy [18]. Furthermore, recently they were used as powerful stimuli-responsive nano-theranostics tools for simultaneously enhancing the accuracy and efficiency of cancer therapies [16]. There is a lot of room to explore CaCO_3_ micro- and nanoparticles in combination with additives or other materials such as polymers, surfactants, liposomes and micelles [19].

The majority of the available pharmaceutical anticancer drugs are hydrophobic in nature and there is an urgent need to develop a selective delivery system for these drugs. Lately, novel composite cyclodextrin—CaCO_3_ spherical porous microparticles have been synthesized to deliver hydrophobic drug [20]. The strategy proposed here could be useful to enhance the bioavailability of many insoluble drugs, considering that approximately 60–70% of the drug molecules are insufficiently soluble in aqueous media and/or have very low permeability [21].

NVP-BEZ235, an imidazo[4,5-*c*]quinoline derivative, efficiently blocks the dysfunctional activation of the PI3K/mTOR pathway in cellular and in vivo settings, thus inhibiting the growth and proliferation of various cancer cells [22,23]. The PI3k/Akt/mTOR pathway controls gene expression related to cell proliferation, differentiation and apoptosis [24]. The prevalence of PI3K signaling abnormalities in human cancer cells has suggested the potential use of PI3K pathway modulators as novel targeted therapeutic agents. Over the past few years, the interests of pharmaceutical chemistry have been directed to develop pan- or isoform-selective PI3K inhibitors with improved pharmacologic characteristics as well as selectivity profile [25,26].

Its very poor solubility in water and ethanol has caused difficulty in managing the variable NVP-BEZ235 toxicity in trials beyond phase I/Ib [27]. Carrier-based drug delivery is a promising approach to improve the bioavailability while at the same time providing the necessary protection of drug molecules [28]. 

As most cancer environments exhibit a lower pH than healthy tissues, the pH value-sensitive feature of CaCO_3_ can stably preserve drugs under physiological conditions and selectively release them in the acidic intracellular tumor environment [29,30,31]. When the CaCO_3_ NCs accumulate at the tumor site through the enhanced permeability and retention (EPR) effect, a fast and stable drug release can be triggered in response to extracellular or intracellular stimulus of tumor cells, where pH value is lower than that in the normal tissue. The liquid tumor microenvironment, like the here proposed model, have a constant pH gradient closer to the normal tissue, but a saturated solution of CaCO_3_ has a predicted pKa of about 9, suggesting a slow but constant tendency to dissolution [15]. In the present work, we propose the use of pure CaCO_3_NCs, to deliver NVP-BEZ235, in order to enhance its solubility, penetration and efficacy in T-cell lymphoma HUT78 cell line. 

As already described in previous works, we synthesized this nanomaterial using the spray drying technique, which may be easily usable on a large scale system. Despite the common multi-parametric nanosynthesis the here-adopted process was easily reproducible with a commercial spray dryer (Mini Spray Dryer B-290, Büchi, Milan, Italy) and ready to be scaled up on an industrial plant [6].

In this work, we demonstrated that these CaCO_3_NCs readily penetrate in T-cell lymphoma, and the nano-formulation with NVP-BEZ235 is more efficient, in term of cytotoxicity and biological response, over the same nominal concentration of free drug. Biological activity of encapsulated NVP-BEZ235 increased with the same trend of its solubility, loading efficiency and intracellular penetration.

Thanks to this strategy we have greatly improved the bioavailability and the membrane permeability of the drug within the cell, as demonstrated with the analytical data concerning the determination of the effective intracellular concentration of the drug. 

For this purpose, an approach based on liquid chromatography coupled to mass spectrometry was adopted to quantify the drug loaded inside NCs, inside cytoplasm, or entrapped in the membrane [32]. This method is convenient compared to other conventional ones that are mostly based on fluorescence [33].

Liquid chromatography/tandem mass spectrometry (LC-MS/MS) is a very sensitive method with limits of detection in the femtomolar range when operating in selected reaction monitoring mode (SRM). A new developed LC-MS/MS method was herein applied to demonstrate that NVP-BEZ235, because of its lipophilicity, remains trapped in the cell membrane, not succeeding in performing its pharmacological action. In the case of nano-formulation (CaCO_3_ NCs-NVP-BEZ235) the drug is easily available to exert its bioactivity in the cytosolic environment, where its biological target is localized.

## 2. Results and Discussion

### 2.1. Nanocrystals Synthesis and Characterization

**Figure 1:**
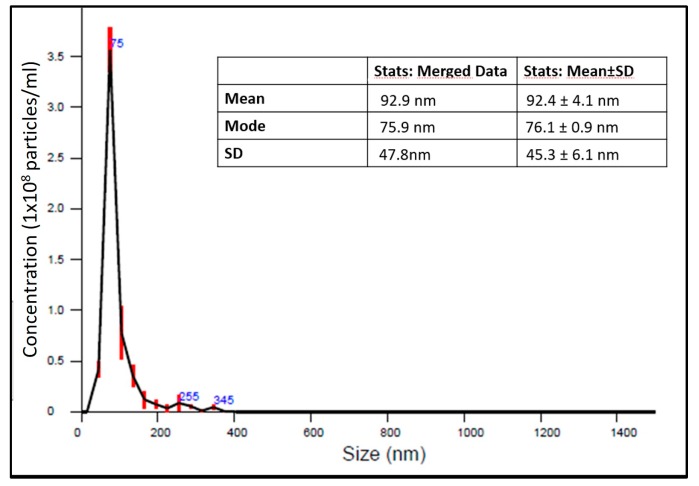
Size distribution and data (table inside) from NTA measurements of CaCO_3_ NCs. Error bars (vertical red lines) represent standard deviations obtained from five measurements of the same sample.

CaCO_3_ NCs were synthesized according to the literature, as described in the Materials and Method section [6,9]. The preparation technique adopted herein allows one to obtain stable calcite nanocrystals which are very advantageous since they do not provoke adverse cellular reactions and do not block the normal cell cycle [34]. CaCO_3_ NCs have an ellipsoidal shape and in order to study their size, together with Transmission Electron Microscopy (TEM) and Dynamic Light Scattering (DLS) measurements (summarized in Table 1) [9], nanoparticle tracking analysis (NTA) was also performed by means of a Nanosight instrument (Malvern, Worcestershire, UK). High resolution particle size, concentration and aggregation measurements under Brownian motion of the NCs suspensions were measured in order monitor in real time the subtle changes in the characteristics of particle populations. The results on size distribution are summarized in Figure 1. CaCO_3_ NCs have a dimension of about 76.1 ± 0.9 nm.

DLS: Dynamic Light Scattering the size measurements were performed at different time point for up to 6 weeks, obtaining always the same results. This result together with ζ-potential measurements suggest that the spray drying synthesis procedure allows to obtain a stable dispersion in water. Furthermore, the Nanosight device provided data about the concentration of CaCO_3_ nanocrystals. In our samples the mean of nanocrystals in 1 mL of ultra-pure water was: 5.54 × 10^9^ ± 3.30 × 10^8^ particles/mL.

### 2.2. Loading of NVP-BEZ235 into CaCO_3_ NCs

The porous morphology of CaCO_3_ is useful to entrap macromolecules efficiently by physical adsorption due to their large surface area [35,36,37]. The loading protocol, as described in the Materials and Methods in Section 3.4, was performed using two different final concentration of the drug: 1 µM and 100 µM. The adsorption of NVP-BEZ235 occurred in the core interstices of CaCO_3_ nanocrystals due to their porous structure: one can argues that the core acts like a sponge that absorbs a proportional amount of drug respect to the solution with whom it comes into contact. The % loading was calculated as reported in the Materials and Methods in Section 3.4. The results indicated that NVP-BEZ235, in both conditions, was completely encapsulated into carbonate nanocrystals (% loading = 99.8 ± 0.1%).

### 2.3. Internalization of CaCO_3_ Nanocrystals in T-Cell Lymphoma Line HUT78

Imaging is a powerful technique for the study of cellular interactions with extracellular substrates, including particles [38,39]. We investigated the internalization by cells of calcium carbonate particles, because the goal of this work is the enhancement of cell penetration of NVP-BEZ235. This drug plays a critical role in the inhibition of PI3K/mTOR pathway competing at its ATP-binding site [40,41,42]. For this reason, it is important that this molecule crosses the cell membrane reaching the cytoplasmic region in order to perform its pharmacological action. Being a poorly water-soluble molecule we thought to deliver it through the CaCO_3_ NCs. First, we checked whether the nanocrystals were internalized in this cell line by confocal microscopy. 

To study uptake, we used the layer by layer (LbL) technique in order to obtain fluorescent CaCO_3_ NCs [9]. The polymers used were PSS (polystyrene sulfonate sodium salt) and PAH (polyallyl amine hydrochloride); only in one of the layers the polyelectrolyte used for coating (labeled as PAH-FITC) was conjugated with fluorescein moieties. 

**Figure 2:**
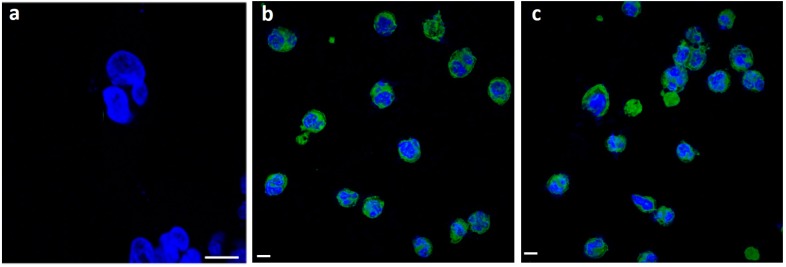
Confocal images of a negative control (**a**), Fluorescein isothiocyanate (FITC)-CaCO_3_ NCs after 5 h (**b**), and 24 h (**c**) in HUT78 cells. In blue are stained nuclei and in green CaCO_3_ NCs. Scale bar, 5 µm.

In particular, the cells were cultured for 5 h with PSS/PAH coated nanocrystals (dosed at a constant particle mass 1.0 mg/mL) since the synthetic multilayer is not enzymatically degraded by proteases. Thanks to their nanometric size and positive superficial charge, internalization by T-cell lymphoma was in fact observed (Figure 2). To minimize the possible interference of the residual nanocrystals externally bound to the cells, a PBS solution was used to wash extensively the cells after incubation.

As shown in Figure 2 fluorescent nanocrystals clearly penetrate the cell membrane and are mainly localized in the cytoplasm. No background fluorescence was observed, after long time incubation period (Figure 2c). Confocal microscopy results showed that the staining pattern of nanocrystals remained the same with a strong fluorescence in the cytoplasm. In particular, no fluorescence was observed in extracellular regions, indicating that nanocrystals internalized within the cells did not leak.

### 2.4. Cytotoxicity Assay

Biological tests in order to verify the effectiveness of the system were assessed. We studied the effects on cell proliferation of 1.0 mg naked CaCO_3_ NCs, free-NVP-BEZ235 (1 μM) and encapsulated NVP-BEZ235 prepared at the final concentration of 1 μM equivalent on T-cell lymphoma line at increasing times (from 30 min up to 48 h) by MTT assay.

**Figure 3:**
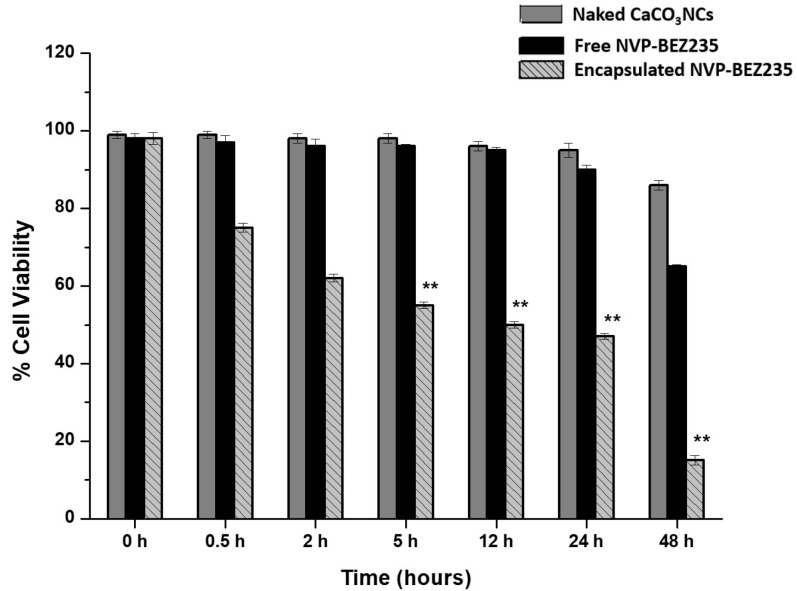
Anti-proliferative effects of naked CaCO_3_ NC (plain gray bars), free NVP-BEZ235 1 μM (black bars) and encapsulated NVP-BEZ235 1 μM equivalent (gray pattern bars) on T-cell lymphoma line (HUT78) at different time points. Values represent mean + SD, and were obtained from three independent experiments. Statistically significant value *p* < 0.05 (*) and statistically highly significant *p* < 0.001 (**).

In Figure 3 the values represent mean ± SD., and were obtained from three independent experiments. The results suggested that encapsulated NVP-BEZ235 was significantly more efficient than free-NVP-BEZ235 at the same nominal concentration with a difference statistically significant. Naked CaCO_3_ NCs have no cytotoxic effects. The cell proliferation evidence suggested that our nano-formulation improved the cell penetration of NVP-BEZ235 leading to an improved biological activity, as confirmed by the quantification of drug performed by LC-MS/MS method.

### 2.5. Quantification of NVP-BEZ235 by LC-MS/MS Method

Free NVP-BEZ235 and encapsulated NVP-BEZ235 (1 μM equivalent) were incubated individually with a T-cell lymphoma line for 2, 5, 12 and 24 h. Due to the very low concentration of the drug and the high complexity of the cell lysate matrix, it was important to develop a highly sensitive analytical method for the detection and quantification of NVP-BEZ235. The choice of the extraction solvent is also crucial, since it affects the amount of drug extracted. Indeed, we used two different extraction solvents and compared the results obtained. First, the analyte was extracted from cell lysate with a hypotonic buffer. Secondly, we performed an extraction with tert-butylmethyl ether (TBME) as described in the Materials and Methods section. In both experiments internalization kinetics of NVP-BEZ235 in a T-cell lymphoma line evaluated by LC-MS/MS was consistent with the biological results, which suggested that nano-encapsulated NVP-BEZ235 was 100 times more efficient than free NVP-BEZ235 (MTT assay). These findings indicated that the use of CaCO_3_ NCs dramatically increased the cell permeation of the drug.

**Figure 4:**
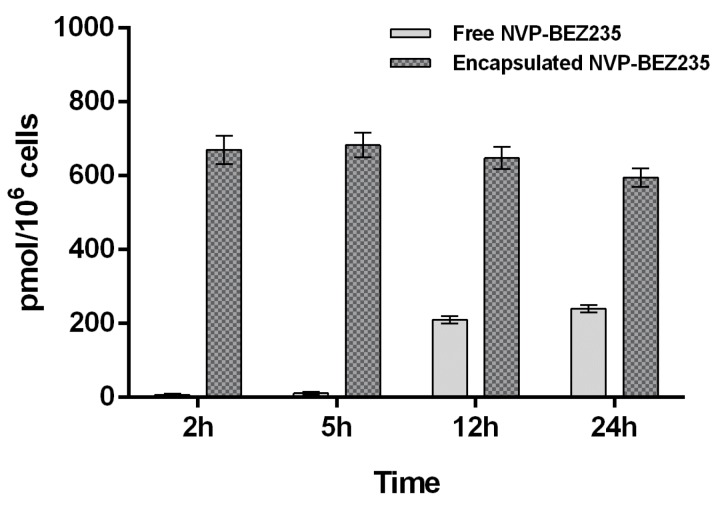
Comparison of intracellular concentrations of NVP-BEZ235 (in the cytosolic fraction) after exposure of T-cell lymphoma line for 2, 5, 12 and 24 h to the free drug (plain gray) and nano-encapsulated drug (gray pattern). The bars are plotted as mean and error bars represent the SD. (*n* = 3).

The results obtained by extracting free NVP-BEZ235 and encapsulated-NVP-BEZ235 from cell lysate with LC-MS/MS mobile phase (acetonitrile:water:formic acid 95:5:0.1 (*v*/*v*/*v*)) are reported in Figure 4.

**Figure 5:**
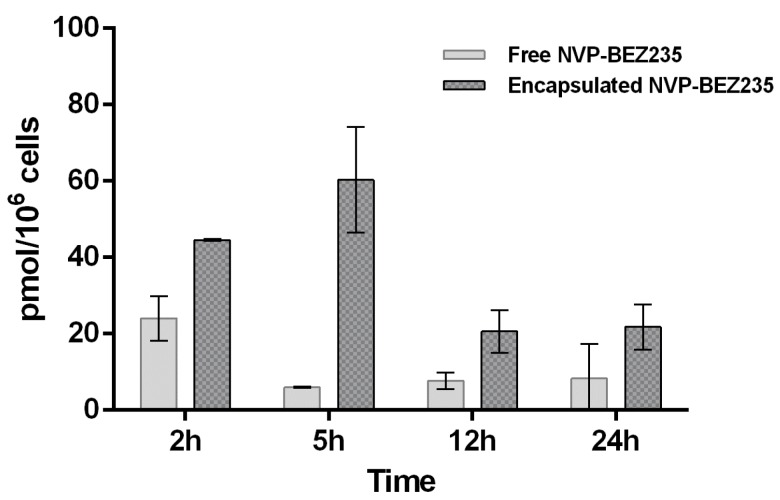
Comparison of vesicular concentrations of NVP-BEZ235 (extracted with TBME) after exposure of T-cell lymphoma line for 2, 5, 12 and 24 h to the free drug (plain gray) and nano-encapsulated drug (gray pattern). The bars are plotted as mean and error bars represent the SD (*n* = 3).

The results, shown in Figure 4 and Figure 5, suggested that the uptake of free NVP-BEZ235 in the cells occurred in the first hour of incubation. After being stored in vesicles, it slowly diffused in the cytosolic fraction over the next 24 h, reaching a concentration of about 0.23 nmol/10^6^ cells. On the other hand, the uptake of nano-encapsulated NVP-BEZ235 in the cytosolic fraction occurred during the first 2 h of incubation reaching the concentration of about 0.67 nmol/10^6^ cells and remaining quite stable over about 12 h.

Furthermore, a different LC-MS/MS method was developed to quantify NVP-BEZ235 entrapped in cell membrane (vesicular fraction). The extraction of NVP-BEZ235 from cell lysate was performed with TBME, which is able to dissolve not only the cytosol content but also that of the cell membranes.

The same LC-MS/MS analyses were then carried out on these new extracted samples in order to understand whether the drug is trapped by the membranes. Indeed, the graph of Figure 5 shows that in the first 2 h free NVP-BEZ235 is mainly concentrated in the cell membrane (0.025 nmol/10^6^ cells). This is most certainly due to its high lipophilicity.

These findings indicated that CaCO_3_ NCs allow to overcome the limitation related to hydrophobicity of this promising drug. These data are confirmed also by western blot assays used to study the dual inhibition pathway (PI3K/mTOR) elicitated by NVP-BEZ235, explained in the next section.

### 2.6. Western Blot Analysis

**Figure 6:**
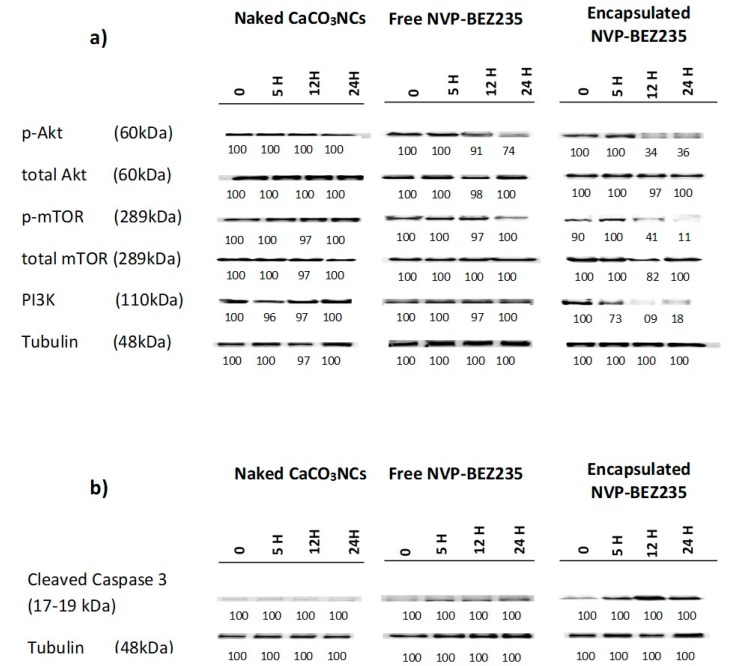
Western blots of cellular extracts from HUT78 cell line treated with naked CaCO_3_ NCs, free NVP-BEZ235 (1 μM) and encapsulated NVP-BEZ235 (1 μM equivalent) for 5, 12 and 24 h. (**a**) Lysates were probed with antibodies against the cleaved form of caspase 3 (Asp175); (**b**) Lysates were probed with antibodies against PI3K, p-Akt (Ser473), total Akt, p-m-TOR (Ser2448) and total m-TOR (Ser2448). Densitometric quantification of bands normalized to the untreated control is shown below the immunoblot bands.

We investigated the effects of 1.0 mg of naked CaCO_3_ NCs, free-NVP-BEZ235 1 μM and encapsulated NVP-BEZ235 1 μM equivalent for 5, 12 and 24 h in targeting PI3K/Akt/mTOR pathway in T-cell lymphoma line. Cellular extracts were probed with antibodies against PI3K, p-Akt (Ser473), total Akt, p-m-TOR (Ser2448) and total m-TOR (Ser2448). Encapsulated NVP-BEZ235 treatment affected the phosphorylation status of p-Akt and p-m-TOR in a time dependent manner (Figure 6a).

The same cellular extracts were probed with antibodies against the cleaved form of caspase 3 (Asp175) to evaluate the apoptotic effects of empty capsules, free-NVP-BEZ235 (1 μM) and encapsulated NVP-BEZ235 (1 μM equivalent) for 5, 12 and 24 h (Figure 6b). 

The encapsulated drug, on the one hand, increases in a time dependent manner the cleaved form of caspase 3, involved in the apoptotic pathway, and on the other side, decreases the phosphorylation status of p-Akt, PI3K and p-m-TOR, involved in cell proliferation and differentiation, in a time dependent manner, more efficiently than the same nominal concentration of free drug. These interesting results could allow to use clinically a dose of encapsulated drug 100 times lower than the dose of free drug, obtaining the same results, thus eliminating in this way all the side effects linked to the intrinsic toxicity of the drug.

## 3. Materials and Methods

### 3.1. Materials and Reagents

The sources of the chemicals are as follows: calcium chloride dehydrate 99.99% (CaCl_2_·2H_2_O, Aldrich, Darmstadt, Germany), sodium hydrogen carbonate (NaHCO_3_) (pro analysis, Merck, Darmstadt, Germany), fetal bovine serum (FBS, Sigma, Darmstadt, Germany), penicillin-streptomycin solution (Sigma), sodium pyruvate (Sigma), RPMI medium (Sigma), thiazolyl blue tetrazolium bromide ≥ 97.5% TLC (Sigma), phosphate buffered saline, Dulbecco A (PBS, Oxoid, Thermo Fisher Scientific, Waltham, Massachusetts, USA), PSS (polystyrene sulfonate sodium salt, Sigma) and PAH (polyallyl amine hydrochloride, Sigma). Dimethylsulfoxide (DMSO; Euroclone, Milan, Italy). NVP-BEZ235 was purchased from Selleck Chemicals (Houston, TX, USA). The internal standard (IS) 5-(2,6-di-4-morpholinyl-4-pyrimidinyl)-4-(trifluoromethyl)-2-pyridinamine (BKM120) was purchased from Selleck Chemicals. The cutaneous T-cell lymphoma line HUT78 was purchased from Deutsche Sammlung von Mikroorganismen und Zellkulturen GmbH (Leibniz-Institute, Germany) and were characterized as specified (https://www.dsmz.de/research/human-and-animal-cell-lines.html). MTT cellTiter nonradioactive cell proliferation assay was from Promega Madison (Fitchburg, WI, USA).

### 3.2. Nanocrystals Synthesis

Pure CaCO_3_NCs were synthesized by an atomization process, as already described in our recent paper [9]. Briefly, the synthesis involves the mixing of two aqueous solutions of NaHCO_3_ and CaCl_2_ at a 2:1 molar ratio, in atmosphere and temperature-controlled environment. The two solutions are mixed using two pumps, which allow a fine control of the flow rate of each reagents. Then they are atomized in a hot air flow at 140 °C. The high temperature causes a rapid evaporation of the aqueous reaction mixture, and allows the direct production of the powder of calcium carbonate and of sodium chloride, which are accumulated in a collection vessel; the reaction by-product (NaCl) can be easily removed by means of later water washings.

### 3.3. Nanocrystals Characterization

The NanoSight NS300 Instrument (Malvern) provides an easy-to-use, reproducible platform for nanocrystals characterization. The NanoSight NS300 uses the technology of Nanoparticle Tracking Analysis (NTA). This unique technology utilizes the properties of both light scattering and Brownian motion in order to obtain the size distribution and concentration measurement of particles in liquid suspension. All measurements were performed at room temperature. The software used for capturing and analyzing the data was the NTA 3.1 Build 3.1.46. The samples were measured for 60 s with manual shutter and gain adjustments. The error bars displayed on the NTA graphs were obtained by the standard deviation of five different measurements of each sample. The mean size and SD values obtained by the NTA software correspond to the arithmetic values calculated with the sizes of all the particles analyzed by the software.

### 3.4. Loading of NVP-BEZ235 into CaCO_3_ Nanocristals

NVP-BEZ235 was dissolved in DMSO to create a 10^−2^ M stock solution that was stored at −80 °C. The stock solutions were further diluted with cell culture medium to appropriate concentrations before use. The maximum final concentration of DMSO (<0.1%) did not affect cell proliferation and did not induce cytotoxicity on the tested cell lines and primary cells (data not shown). The protocols to load this drug were the following: to a aqueous suspension containing 1.0 mg of naked calcium carbonate nanocrystals was added a quantity of the drug from stock solution in order to obtain two final concentration of NVP-BEZ235 of 1 µM and 100 µM, followed by stirring of the mixtures for 24 h at room temperature. Unbound drug was removed by dialysis against deionized water for 5 h using membrane with MWCO 3500. After washing and centrifugation, drug loading was calculated in the supernatant by LC-MS/MS by the following formula:

%loading = 100 − ([NVP-BEZ235] prior loading − [NVP-BEZ235] after loading)/[NVP-BEZ235] prior loading

The concentration of NVP-BEZ235 prior loading was 100 μM and the concentration after loading was calculated from the area of the chromatographic peak of the analyte by LC-MS/MS.

### 3.5. Cell Culture

HUT78 cell line was cultured in RPMI-1640 supplemented with 10% fetal bovine serum (FBS, heat inactivated at 56 °C for 30 min), 2 mM L-glutamine and 100 U/mL penicillin and streptomycin, under normal conditions (37 °C, 5% CO_2_ under humidified atmosphere) in suspension flasks. 

### 3.6. Cell Proliferation Assay

T-cell lymphoma line was treated with free NVP-BEZ235 (1.0 µM) and encapsulated NVP-BEZ235 (1.0 µM equivalent) at different times. Cell proliferation was evaluated by MTT assay, which measures the conversion of a tetrazolium compound into formazan by a mitochondrial dehydrogenase enzyme in live cells. Briefly, 15 μL of MTT [3-(4,5-dimethylthiazol-2-yl)-2,5-diphenyltetrazolium bromide] was added to each well for 4 h at 37 °C. A solubilizing solution was added for 1 h, and the absorbance was then measured at 550/630 nm using a plate reader (DAS, Rome, Italy). The relative cell viability was expressed as a percentage of the untreated control wells. Each data point is the average of three independent determinations. A *p* values of <0.05 were considered statistically significant.

### 3.7. Confocal Laser-Scanning Fluorescence Microscopy (CLSM)

Laser scanning confocal microscopy was performed on a Zeiss LSM700 (Zeiss, Goettingen, Germany) confocal microscope equipped with an Axio Observer Z1 (Zeiss) inverted microscope using an objective 100×, with 1.46 numerical aperture oil immersion lens for imaging. Laser beams with 405 and 488 nm excitation wavelengths were used for Hoechst dye and labelled CaCO_3_ NCs, respectively. Cancer cell lines (5 × 10^4^) were seeded onto 35 mm glass bottom Petri Dish and incubated over-night. The cells were incubated with the fluorescent CaCO_3_ NCs for 5 h in the dark in a humidified incubator at 37 °C, 5% CO_2_, and 95% relative humidity. The cells were then rinsed with PBS and fixed with glutaraldehyde 0.25%. All samples were stained with Hoechst 33342 (0.2 μM) for 5 min to check the nuclei morphology. The images were examined under confocal microscope.

### 3.8. Western Blot Analysis

After drug treatments, the cell lines were harvested and lysed using the Mammalian Cell Extraction Kit (BioVision, Milpitas, CA, USA) following the manufacturer’s instructions. Proteins (100 μg/lane) were electrophoresed on 4–20% SDS-polyacrylamide gradient gels and transferred to nitrocellulose membranes (Bio-Rad Laboratories, Hercules, CA, USA). Membranes were immunoblotted with the following primary antibodies: total AKT, p-AKT (ser 473), total m-TOR, p-m-TOR (ser 2448) and caspase-3 (Asp175). All of the above-mentioned antibodies were diluted 1:1000 and purchased from Cell Signaling Technology (Danvers, MA, USA). Then, the membranes were incubated with species-specific horseradish peroxidase- (HRP-) conjugated secondary antibody (1:500) from Cell Signaling Technology. Blots were developed using SuperSignal West Pico Chemiluminescent Substrate (Thermo Scientific, Rockford, IL, USA). Images were acquired by Chemidoc XRS.

### 3.9. Preparation of Standard Solutions

Individual stock solutions of analyte (NVP-BEZ235) and IS (BKM120) were prepared in DMSO to yield the final concentration of 1 mg/mL. Further dilutions of NVP-BEZ235 were prepared in blank cell lysate to yield the calibration samples at 1.00, 5.00, 10.0, 50.0 and 100 μM. Independently prepared NVP-BEZ235 stock solutions at 2.5, 25 and 75 μM were used as low concentration quality control (LQC), medium concentration quality control (MQC) and high concentration quality control (HQC) samples, respectively. QC samples were prepared as for calibration standards. 1.0 mg/mL stock solution of BKM120 was further diluted with acetonitrile (ACN) and added to each calibration sample to get a 1.0 μg/mL final concentration. Calibration samples, QC samples and IS working solution were aliquoted and stored at −20 °C.

### 3.10. Sample Preparation of Cell Lysates

#### 3.10.1. Extraction of the Cytosolic Fraction

HUT78 cancer cells were individually incubated with a solution 100 μM free NVP-BEZ235 and encapsulated NVP-BEZ235 100 μM equivalent (preparation reported above) at 37 °C for 2, 5, 12 and 24 h. Cultured medium was removed and cells were twice washed with ice-cold acid PBS (phosphate buffered saline, pH 5.0), scarpered, counted and centrifuged. Each cell aliquot (1 × 10^6^ cells) was lysed by 25 µL of hypotonic buffer (pH 8, 20 mM HEPES, 10 mM KCl, 1.5 mM MgCl_2_, 1 mM EDTA, 250 mM sucrose, 0.1 mM PMSF, containing protease inhibitor cocktail), added of IS (final concentration 1 µg/mL) and acetonitrile (25 µL), thermostatted at 95 °C for 5 min and centrifuged at 20,817× *g* for 30 min. The supernatants were dried, reconstituted with 25 µL of mobile phase and injected into the LC-MS/MS instrument. 

#### 3.10.2. Extraction of the Vesicular Fraction

The pellets were suspended in a solution of IS (final concentration 1 µg/mL) and *tert*-butylmethyl ether (TBME) (25 µL). The suspension was mixed and centrifuged for 5 min at 20,817× *g*. The supernatants were dried, reconstituted with 25 µL of mobile phase and injected into the LC-MS/MS instrument.

### 3.11. LC-MS/MS Method for the Quantification of NVP-BEZ235 in Cell Lysates 

The quantitative analyses were carried out on an Agilent 1200 liquid chromatograph (Agilent Technologies, Milan, Italy) consisting of a binary pump, an autosampler and a thermostated column compartment. An Agilent 6410 triple quadrupole-mass spectrometer (Agilent Technologies) with an electrospray ionization source operating in positive mode was used for detection (LC-ESI-QqQ). Flow injection analyses of the analyte and IS were performed to optimize the fragmentor and source parameters, which were: gas temperature 350 °C, gas flow 10 L/min, nebulizer 32 psi, capillary voltage 4500 V, fragmentor 135 V. In order to determine the characteristic mass fragments for multiple reaction monitoring (MRM) analysis, the product spectra of NVP-BEZ235 and IS were recorded in full scan mode by varying the offset voltage between 10 and 45 eV. The optimal collision energy value was 45 eV. The quantitative analyses were carried out using selected reaction monitoring (SRM) following the reactions: *m*/*z* 470.4 → 443.3 and *m*/*z* 411.3 → 367.3 as quantifiers for NVP-BEZ235 and IS, respectively; *m*/*z* 470.4 → 454.3 and *m*/*z* 411.3 → 307.2 as qualifiers for NVP-BEZ235 and IS, respectively. Mass spectrometry chromatograms were acquired and analyzed using Agilent MassHunter Qualitative Analyses Version B.01.04 data processing software. The analyses were performed on a Poroshell 120 (50 × 3.0 mm I.D., 2.7 μm) with a mobile phase composed of acetonitrile:water:formic acid 95:5:0.1 (*v*/*v*/*v*) at a flow rate of 0.5 mL/min. The injection volume was 5 µL.

### 3.12. LC-MS/MS Method Validation

The LC-MS/MS method developed was validated for sensitivity, specificity, linearity, accuracy, precision, recovery, matrix effect and stability. The method validation details are reported in Appendix A.

### 3.13. Statistical Analysis

Statistical differences between controls and drug-treated cells were determined by one-way ANOVA (Sidak). *p*-values < 0.05 were considered statistically significant. Data were analyzed using the Stata 8.2/SE package (StataCorp LP, College Station, TX, USA).

## 4. Conclusions

It is well established that drug tolerance is one of the key challenges for dual PI3K–mTOR inhibitors as NVP-BEZ235, which explains the slow progress of research and development of these inhibitors. Currently, only eleven new dual PI3K–mTOR inhibitors, including NVP-BEZ235, are being evaluated actively in the clinical stage, is currently in phase I/II clinical trials for advanced solid tumors (ClinicaTrials.gov, U.S. National Institutes of Health, Bethesda, MD, USA: http://clinicaltrials.gov), but none has been approved by the FDA. Although significant therapeutic effects against cancer have been demonstrated, NVP-BEZ235 have shown significant toxicity, including stomatitis, noninfectious pneumonitis, rash, hyperglycemia, and immunosuppression. Encapsulation strategy offers a potential approach for enhancing the solubility of NVP-BEZ235 improving its bioavailability and suggests the potential use of this formulation for oral administration. Oral administration would allow outpatient treatment, improving quality of life and reducing treatment costs.

Cytosolic delivery strategies could contribute to simplify the entry or export of various cargo that are insufficiently permeable to plasma membrane. In this work we proposed an engineered system, based on CaCO_3_, to deliver NVP-BEZ235. Because of their central role in the initiation and progression of tumors, blockade of the mTOR and PI3K signaling pathways has emerged as a compelling target for the development of novel drug formulations for maximal therapeutic efficacy in many tumors. Taken together, it appears that dual PI3K/mTOR inhibitors, such as NVP-BEZ235, are required. Because of its intrinsic chemical hydrophobicity, NVP-BEZ235 is poorly tolerable from patients and negatively influenced the clinical studies [27]. Its toxicity profile causes high percentage of adverse events leading to frequent discontinuation treatments. Despite these problems NVP-BEZ235 remains an attractive agent. Therefore, in order to overcome its chemical limitations, it is necessary to either reduce the administered dose or combining the efficacy of this molecule with other drugs for a synergic action. In view of its potentiality, and on the other side of its limitations, we focused this work on finding a nanotechnological strategy to achieve higher efficacy of the drug toward cancer cells, decreasing drug concentration and increasing drug penetration. Hence, we developed at first a promising payload drug delivery system based on CaCO_3_ NCs that improved the water solubility of NVP-BEZ235 making it immediately available to cell machinery. Furthermore, a new highly sensitive analytical method methods have been used to determine, qualitatively or quantitatively, whether NVP-BEZ235 has successfully traversed the cell membrane. The obtained nano-formulation has a cytosolic localization more efficient (almost 30%) than the free drug as demonstrated in the quantification analysis of NVP-BEZ235 in the intracellular fraction. This result is affected in a cytotoxic effect almost 50 times higher than free drug at the same nominal concentration, in T-cell lymphoma line, as well as in the phosphorylation of proteins involved in the mTOR pathway, as demonstrated in the western blot analysis. These overall results show that the fabrication of loaded NVP-BEZ235 CaCO_3_ nanocrystals is a promising drug delivery system able to improve the pharmacokinetics of this drug.

## Figures and Tables

**Table 1:** Size measurements [9].

TEM Measurement (nm)	DLS Measurement (nm)	ζ-Potential Measurement (mVolt)
Major diameter 93 ± 9	UP water: 222.7 ± 9.2	UP water: 6.8 ± 0.1
Minor diameter 35 ± 10	PBS: 188.8 ± 4.4	PBS: −25.1 ± 0.3
	Culture media: 344.0 ± 32.8	Culture media: −11.2 ± 0.2

TEM, Transmission Electron Microscopy; DLS, Dynamic Light Scattering.

## Appendix A. LC-MS/MS Method Validation

• Detection and quantification limits (sensitivity)

Limit of quantification (LOQ) was defined as the concentration at which the quantifier transition of BEZ235 yielded a signal to noise ratio (S/N) of 10 and it was 1 μM in cell lysate. Limit of detection (LOD) in cell lysate was estimate based on 5:1 S/N and it was 0.5 μM.

• Specificity

Typical SRM chromatograms of BEZ235 and IS in cell lysate showed no interfering peaks from endogenous substances at the retention time of BEZ235 and IS. The chromatograms of blank cell lysate also had no interfering peaks at the retention times of BEZ235 and IS. The specificity results indicate that the method is highly selective for BEZ235 analysis.

• Calibration curve and Linearity

Calibration curves for BEZ235 were performed in cell lysates (1 to 100 μM). Five replicates were used to establish the linear calibration equation (y = mx + c) and analyzed using the ratio of analyte peak area over IS peak area after quantitative integration by Mass Hunter software. Linearity was measured as the coefficient of determination (R2) for five calibration replicates. The acceptance criteria for each back-calculated standard concentration were ±15% deviation from the nominal value except at LOQ, which was set at ±20%. Linearity was excellent in the range 1–100 μM with corresponding coefficient of determination R2 > 0.998.

• Accuracy and precision

Intra-day accuracy and precision were assessed from five replicates of LOQ and QC concentration levels (low LQC, middle MQC and high HQC). Inter-day accuracy and precision were assessed on three triplicates for five-day analysis of the three QC concentration levels. Accuracy of the method was calculated as percent deviation based on the difference between the mean concentration found and the nominal concentration. The percent deviation should be within 15% of the actual value, except at the LOQ, where it should not deviate by more than 20%. Precision was evaluated as percentage coefficient of variation (%CV) of the mean concentration found. The precision determined at each concentration level should not exceed 15% of the CV, except for the LOQ, where it should not exceed 20% of the CV.

The accuracy and precision data for cell lysate. The intra-day accuracy showed percent deviation ranging from −1.19 to 4.22% with a %CV from 0.88 to 4.65%. The inter-day accuracy and precision showed percent of deviation ranged from to −3.80 to 8.50% with a %CV from 2.09 to 8.78. Since the accuracy and precision values were found within the acceptance limits, these results suggested that the method is accurate and precise.

• Recovery

The extraction recovery of BEZ235 was determined at the LQC, MQC and HQC levels by comparing the peak areas of BEZ235 extracted from spiked cell lysate samples prepared by spiking BEZ235 in blank cell lysate with the corresponding concentration of authentic standard solutions. Experiments were performed in five replicates. The extent of recovery should be consistent, precise and reproducible. The recovery of extraction of BEZ235 from cell lysate was >82%.

• Matrix effect

The matrix effect was evaluated by determining the matrix factor (MF) and the IS-normalized MF from cell lysate. The determination was performed at the LQC, MQC and HQC levels in triplicate. The MF of BEZ235 was calculated by comparing the peak area of BEZ235 extracted from samples prepared by spiking BEZ235 in cell lysate with the corresponding concentration of the authentic solution of BEZ235 prepared in mobile phase. The MF of IS was determined and calculated in a similar manner. The IS-normalized MF is the ratio between the MF of BEZ235 and that of IS. The %CV of the IS-normalized MF from blank cell lysate should not exceed 15%. The %CV of IS-normalized MF in cell lysate was <10%. The %CV < 15% suggests that ion suppression or enhancement from cell lysate matrix is negligible in the assay.

• Stability

Stock solutions of BEZ235 and IS were checked for short-term stability at room temperature, autosampler stability at 10 °C, freeze-thaw cycles at −20 °C and long-term stability at −70 °C. Stability results in cell lysate were evaluated by measuring the area ratio response (analyte/IS) of stability samples against freshly prepared comparison standards at the same concentration. The solutions were considered stable if the deviation was within ±10%. Tests for bench top at room temperature, autosampler stability at 10 °C, freeze-thaw cycles at −20 °C and long-term stability at −70 °C were performed using six replicates. The stability samples were quantified against freshly prepared comparison standards at the same concentration. Stability data were acceptable if the %CV of the replicate determinations did not exceed 15% and the mean accuracy value was within ±15% of the nominal value. Short-term stability analysis for bench top at room temperature and autosampler at 10 °C showed %CV lower than 9.8% and 4.2%, respectively, indicating that BEZ235 is stable under experimental conditions. %CV after three freeze-thaw cycles at −20 °C and for long-term at −70 °C were >90% and >50%, respectively, indicating low stability of BEZ235 in these conditions.
